# The landscape of chimeric RNAs in non-diseased tissues and cells

**DOI:** 10.1093/nar/gkz1223

**Published:** 2020-01-22

**Authors:** Sandeep Singh, Fujun Qin, Shailesh Kumar, Justin Elfman, Emily Lin, Lam-Phong Pham, Amy Yang, Hui Li

**Affiliations:** 1 Department of Pathology, School of Medicine, University of Virginia, Charlottesville, VA 22908, USA; 2 National Institute of Plant Genome Research (NIPGR), New Delhi 110067, India; 3 Department of Biochemistry and Molecular Genetics, School of Medicine, University of Virginia, Charlottesville, VA 22908, USA

## Abstract

Chimeric RNAs and their encoded proteins have been traditionally viewed as unique features of neoplasia, and have been used as biomarkers and therapeutic targets for multiple cancers. Recent studies have demonstrated that chimeric RNAs also exist in non-cancerous cells and tissues, although large-scale, genome-wide studies of chimeric RNAs in non-diseased tissues have been scarce. Here, we explored the landscape of chimeric RNAs in 9495 non-diseased human tissue samples of 53 different tissues from the GTEx project. Further, we established means for classifying chimeric RNAs, and observed enrichment for particular classifications as more stringent filters are applied. We experimentally validated a subset of chimeric RNAs from each classification and demonstrated functional relevance of two chimeric RNAs in non-cancerous cells. Importantly, our list of chimeric RNAs in non-diseased tissues overlaps with some entries in several cancer fusion databases, raising concerns for some annotations. The data from this study provides a large repository of chimeric RNAs present in non-diseased tissues, which can be used as a control dataset to facilitate the identification of true cancer-specific chimeras.

## INTRODUCTION

Over the past decade, there has been tremendous development in the field of chimeric RNA discovery, pursuing identification of novel biomarkers and therapeutic targets in cancer (1–4). Specifically, RNA-seq data availability from large projects such as TCGA (5) allows for high throughput discovery of chimeric RNAs in cancer (6–10). Although chimeric RNAs were traditionally thought to be unique features of cancer cells (11), there is also an emergent role for chimeric RNAs in inherited disorders (12,13). Moreover, recent research has validated the existence of chimeric RNAs in non-cancerous cells and tissues (14–20). These findings highlight the need to establish a true baseline of chimeric RNAs in normal physiology before cancer-specific fusion events can be identified.

In our previous work, we integrated data from 300 RNA-seq libraries across 30 non-neoplastic tissues to project a landscape of chimeric RNAs in non-cancer tissues and cells (14). We obtained samples from non-cancer donors (14,21) and found examples of tissue-specific and housekeeping chimeric RNAs (14). However, there are limitations of that study, including: sample selection and curation to ensure proper histology of tissues; sample size; and the stringency of filters for recurrent events.

The Genotype-Tissue Expression (GTEx) dataset represents an ideal resource for non-cancerous chimeric RNA study in that the samples are carefully curated to ensure normal histology (22,23), and the paired-end RNA-seq platform is favored for chimeric RNA data-mining (24–26). In this study, we explore the landscape of chimeric RNAs in 9495 GTEx samples. We examine the potential for chimeric RNA profiling in the characterization of different tissues and present evidence to support the functional relevance of two chimeric RNAs resulting from different splicing mechanisms. We also consider implications of detecting of supposed cancer-specific chimeras in GTEx samples, utilizing the TCGA bladder cancer dataset as a case study to demonstrate the value of GTEx chimeric RNAs as a filter to enrich for true cancer-specific chimeric RNAs.

## MATERIALS AND METHODS

### Data acquisition

RNA-seq data were downloaded from the GTEx project (V6 dbGaP Accession phs000424.v6.p1)(22,23). To check the overlap between GTEx chimeras and cancer-specific chimeras, gene fusion lists from COSMIC (27), Mitelman (28) and TICdb (29) were downloaded. To perform the chimeric peptide identification, mass spectrometry data files in *.mzML format corresponding to 30 colon samples were downloaded from The Clinical Proteomic Tumor Analysis Consortium (CPTAC) resource (30).

### Bioinformatic prediction of chimeric RNAs

Default parameters were used to run EricScript (31,32) with GRCh38 as the reference genome and paired-end RNA-seq fastq files as input. Chimeric RNAs with an EricScore (EricScript prediction score) <0.6 or without predicted breakpoint positions were discarded. Blat (33) was used to apply sequence identity-based filter (identity cutoff 90%) to remove potential false positive chimeras whose junction sequence matched human reference transcriptome from Ensembl (34), RefSeq (35,36) or Gencode (37) annotations. Based on the junction coordinates, chimeric RNAs were classified into E/E (both coordinates of parental genes map to the ends of exons), E/M (coordinate of 5′ and 3′ gene map to the end and the middle of exon respectively), M/E (coordinate of 5′ and 3′ gene map to the middle and the end of exon respectively) and M/M (both coordinates of parental genes map to the middle of exons) (14). Relative expression of chimeric RNA to parental genes was calculated using read count-based expression values provided in the EricScript output. We filtered out the cases where this ratio exceeded 1. To determine whether the chimeric RNA encodes for an in-frame or frame-shifted peptide, we used ‘predict_frame.py’ python script from FusionCatcher software (38).

Tissue-specific recurrent chimeric RNAs were parsed from GTEx (22,23) predictions after removal of M/M designated chimeric RNAs. Tissue-specific recurrent chimeric RNAs were defined as those with a frequency of at least 2 in tissues with a total of 100 or fewer samples, at least 3 in tissues with 300 or fewer samples or at least 5 in tissues with >300 samples.

For examination of GTEx chimeras in COSMIC (27), we searched both the parental genes, and junction sequence locations; For Mitelman (28) and TICdb (29) databases, we searched only the parental gene names, as these two databases do not provide exact breakpoint positions. Gene ontology terms were predicted for the 5′ and 3′ parental genes of chimeras using Gorilla (39) with all annotated genes in hg38 as background and a *P*-value cutoff of <0.001.

### Chimeric RNA profiling

A matrix of samples and unique recurrent chimeras were created, with each cell having a value of either 1 or 0 representing the presence or absence (binary profile) of the chimera in the corresponding sample. We also created binary profile of parental genes and compared with chimeric RNA profile to measure the similarities/dissimilarities among these matrices. To create the binary matrix of parental genes, we first downloaded the gene expression values from GTEx and made separate expression matrix for 5′ genes and 3′ genes of the chimera from GTEx-noMM-recurrent set. Next, we identified the lowest expression value of all the chimeras being FPKM of 0.04. Using 0.04 as cutoff, we then converted the expression values of parental gene matrix to 1 if the value was ≥0.04 else 0. This assures that for all the chimeras, we are treating their parental genes as expressed in a particular sample the same as chimeras. Next, we used Simple Matching Coefficient (SMC) which compares two binary inputs and gives a score from 0 to 1, wherein 0 means no similarity and 1 means identical. For each chimeric RNA binary profile, we calculated SMC score with 5′ as well as 3′ binary profile.

### Motif prediction

To identify motifs present in the 5′ and 3′ parental genes, we used the Gapped Local Alignment of Motifs (GLAM2) tool (40) from the MEME SUITE (41) with default parameters to find enriched motifs in the 200 bp upstream and downstream sequences from the breakpoint position. Further, we used the Tomtom tool (42) from MEME SUITE (41) with default parameters on the list of identified motifs and scanned a database of RNA binding protein motifs (43).

### Chimeric peptide prediction

To identify chimeric peptides using the database search method, we first constructed a database of chimeric peptides from the recurrent chimeras from colon sigmoid and colon transverse tissues in GTEx. The 200 bp upstream and downstream sequences from the breakpoint position were combined together to form the chimeric nucleotide sequence. Three-frame translation of the chimeric sequence was performed using the ‘transeq’ script of the EMBOSS software package (release 6.6.0) (44). *In silico* digestion of the peptides from each frame was performed using the EMBOSS ‘pepdigest’ script using trypsin as the designated enzyme. For each frame, the fragment which spanned the chimeric junction was retained as the chimeric peptide for that frame, provided at least two amino acids were present on either side of the junction. These predicted peptides were stored as a database of chimeric peptides from colon tissue. We expanded this database via inclusion of protein sequences from the neXtProt (45) database to the database of chimeric peptides. We obtained mass spectrometry data files corresponding to 30 colon samples from the CPTAC (30) as a comparative reference for the chimeric predictions.

MGSF+ (46) was run in high-precision mode to obtain peptide spectrum matches (PSMs). FDR values were computed by comparison to a decoy database generated by the MSGF+ package. Chimeric peptides identified from PSMs were considered positive hits if *q* < 0.05 and at least two amino acids were present on each side of the chimeric junction.

### qRT-PCR and sanger sequencing

Chimeric RNA candidates predicted by EricScript (31) were confirmed by qRT-PCR. All RNA samples in this study were treated with DNase I (NEB, M0303), and followed by reverse transcription using the SensiFAST kit (Bioline, BIO-65054). qPCR was performed using the Applied Biosciences StepOne Plus system (Life Technologies) with the SensiFAST SYBR with HiRox reagent (Bioline, BIO-92005). Following qRT-PCR and gel electrophoresis, purified DNA bands were sent to Genewiz for Sanger sequencing. In knockdown experiments, the 2^−ΔCt^ method was used to compare relative RNA expression between samples. All qRT-PCR primers are listed in Supplementary Table S1.

### Cell culture, siRNA knockdown and transfection

Prostate cell line RWPE-1 was maintained in RPMI 1640 medium containing 10% FBS (Fetal Bovine Serum(HyClone)). Immortalized astrocytes were grown in DMEM/F12 with 10% FBS, and supplemented with sodium bicarbonate and glucose. Each media was supplemented with 1% pen/strep and 1% L-Glutamine. All siRNAs were synthesized by Life Technologies and transfected into RWPE-1 and astrocytes with Lipofectamine RNAiMax (Life Technologies) following the manufacturer's instructions. siRNA targeting sequences are: si-negative (−), CGTACGCGGAATACTTCGA; siAN-1, TCCGCCCTTGGTTTCAAAG; siAN-2, GGGTCCGCCCTTGGTTTCA; siCCB-1, TCCGAAGTCAGGAAATATT; siCCB-2, ACATCCGAAGTCAGGAAAT.

### Wound healing and cell counting

RWPE-1 cells and astrocytes were transfected with si-negative, or siRNAs against *ADCK4-NUMBL* or *C15orf57-CBX3*, and were cultured for about 3 days to obtain 80–90% monolayer confluency. A wound was made by scratching the cells using a 10 µl plastic pipette tip, and the medium was immediately replaced. Images were captured immediately after wounding and 10 h later. Cell migration quantified by the size of the wounds in μm. In parallel, cell proliferation was quantified via cell counting in each experimental condition.

## RESULTS

### GTEx chimeric transcriptome

**Figure 1.**
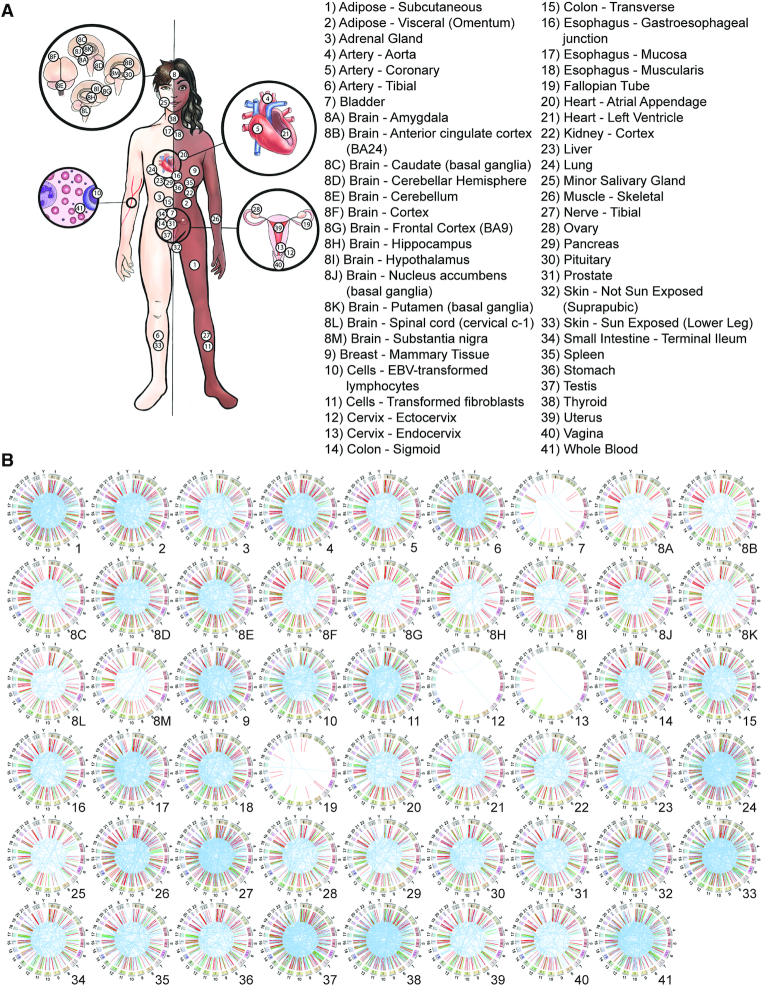
The landscape of chimeric transcriptome. (**A**) Illustration of 53 different adult tissues of human body in the GTEx project. (**B**) The landscape of recurrent chimeric RNAs and their classifications based on parental gene location (red = read-through, blue = inter-chromosomal and green = intra-others) in each tissue. Chimeric transcripts are visualized as a line that connects its two parental genes. Several tissues including bladder, ectocervix and endocervix are represented by fewer samples, and thus exhibit fewer chimeras.

We used the EricScript software package (31) to predict the chimeric RNA profile for each RNA-seq sample from GTEx (23). A total of 2 515 721 chimeric RNAs were predicted from 9495 samples comprising 549 individuals (Figure 1A), which represent 617 880 unique chimeric RNAs. Using blat, we applied sequence identity-based filter to remove 58 517 potential false positive chimeras. This filtering step eliminated the chimeras whose junction sequence is highly similar to reference transcript in Ensembl (34), RefSeq (35,36) or Gencode (37), resulting in a total of 559 363 unique chimeric RNAs (Supplementary Table S2). Out of all unique chimeras, 180 924 were predicted to possess junction sequences at the ends of annotated exons (E/E) or at the end of one annotated exon and in the middle of another annotated exon (M/E, E/M), and 14 114 chimeras were detected five or more times within our dataset. These designations have shown to be important distinguishing criteria for elimination of false-positive predictions; chimeric RNAs belonging to the M/M category exhibit low experimental validation rates (47) and may represent artifacts such as template switching during library construction (14). The last dataset contains a total of 7193 unique gene-pairs, with 4579 unique 5′ parental genes and 4920 unique 3′ parental genes (Supplementary Table S3). The representation of different tissues and the landscape of these recurrent chimeras in GTEx are shown in Figure 1.

### Chimeric RNA profiling

**Figure 2.**
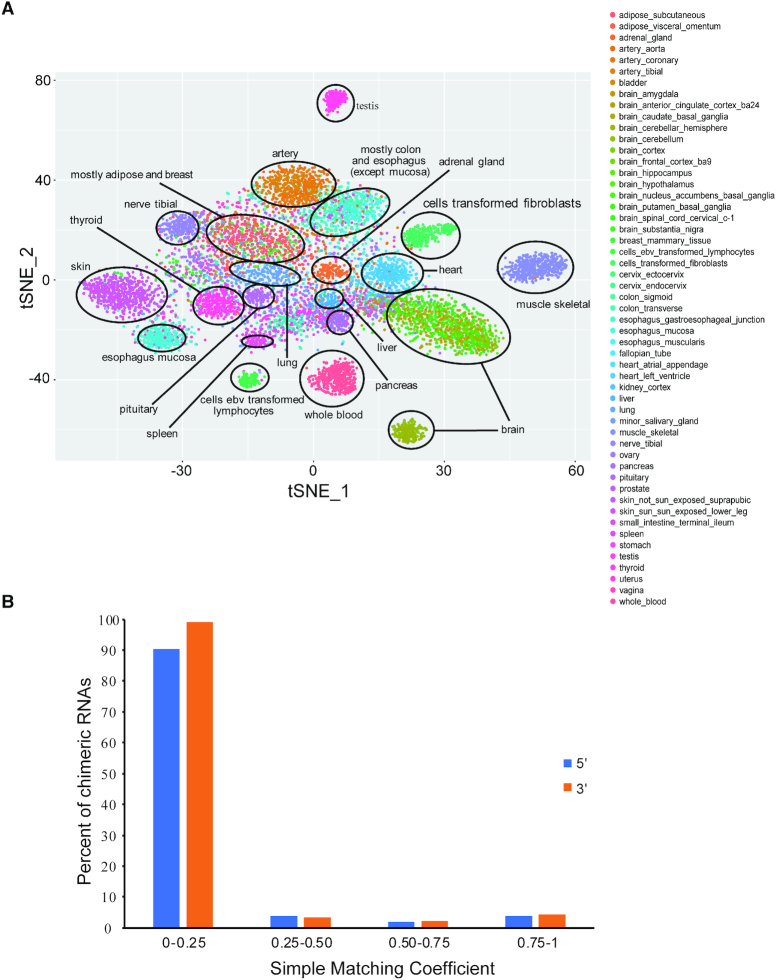
(**A**) t-SNE plot of GTEx samples using a binary profile of chimeras. The presence or absence of chimeric RNAs was translated into a matrix of 0 or 1 for each chimeric RNA. Rtsne package was used to generate a t-SNE plot. Twenty-one unique clusters were identified. (**B**) Distribution of SMC scores after comparison between chimeric RNAs and their parental genes using their binary profiles. The presence or absence of chimeric RNAs was translated into a matrix of 0 and 1 for each chimeric RNA. For parental genes of the chimeric RNA, a matrix of 0 and 1 was also created using their expression profile based on a cut-off score of 0.04 (lowest expression value of any chimeric RNA in Non-M/M-Recurrent set). SMC score was calculated for the binary profiles of chimeric RNAs and their parental genes.

The fact that chimeric RNAs have been used as differential diagnostic markers in cancer suggests that they may be tightly associated with specific cell types or tissue differentiation lineages. Recently, we demonstrated that chimeric RNA profiling can be used to group biological samples and reveal similar expression patterns between seemingly unrelated samples (48). We created chimeric RNA profiles by designating their presence or absence (binary profile) in each GTEx sample and used t-Distributed Stochastic Neighbor Embedding (t-SNE) to visualize unbiased clustering of samples. Samples with similar cell or tissue of origin together were grouped together on t-SNE plot (Figure 2A). A total of 20 clusters can be identified, including testis, whole blood, lymphocyte, skin, esophagus mucosa, pancreas, liver, heart, adrenal gland, lung, colon, spleen, pituitary, thyroid, nerve tibial, artery, adipose, and breast, muscle skeletal, brain and fibroblast (Figure 2A). We also created binary matrix for parental genes, but failed to run t-SNE analysis due to too many identical rows and columns. To quantify the similarity/difference between chimeric RNA profile and those of the parental genes, we calculated SMC score of the profiles of chimeric RNAs and parental genes. SMC score ranges from 0 to 1 with 0 meaning no similarity and 1 meaning identical. We observed that ∼90% of the chimeric RNAs are dissimilar as compared to the 5′ and 3′ parental genes (SMC 0–0.25). Only ∼4% fusions had high similarity with their parental genes (SMC ≥ 0.6) (Figure 2B). Therefore, chimeric RNA expression profile is mostly different from the profiles of their parental genes. In contrast to the canonical transcriptome, where expression is used as a quantitative trait, chimeric RNAs may potentially be viewed as qualitative traits.

### Characterization and distribution of chimeric RNAs

**Figure 3.**
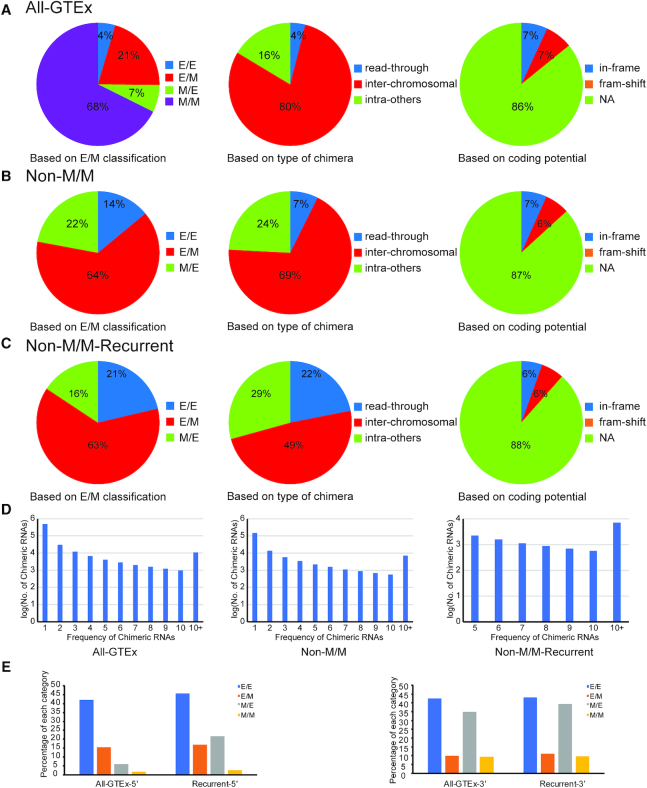
Distribution of chimeric RNAs in different EM categories, types of chimeras based on parental gene location, and fusion protein coding potential. The distribution of chimeric RNAs was examined at three stages along our filtering pipeline: All GTEx predictions (All-GTEx) (**A**), after removal of M/M (Non-M/M) (**B**), and with an additional frequency requirement (Non-M/M-Recurrent) (**C**). The number of chimeric RNAs is also plotted based on their frequency (**D**). (**E**) Percentage of chimeric RNAs harboring the canonical splicing donor sequence (AG/GT) at the 5′ junction (left) or canonical splicing acceptor sequence (AG/G) at the 3′ junction (right) is plotted. All four categories of chimeric RNAs (E/E, E/M, M/E and M/M) in the whole GTEx or recurrent groups were examined.

Chimeric RNAs were characterized based on fusion junction site, parental gene location and fusion protein coding potential as previously described (14,47). Each candidate chimeric RNA was designated a parental gene classifier (inter-chromosomal, read-through, intra-others) based on parental gene chromosomal location, proximity and orientation, as well as a chimeric junction classifier of E/E, E/M or M/E as previously described (14). In addition, based on the reading frame of the two parental genes when the fusion is formed, we categorized the chimeras into ‘in-frame’, ‘frame-shift’ and ‘NA’. We then examined the distribution of these chimeric RNAs through three filtering stages: inclusion of all predicted chimeric RNAs (All-GTEx); after elimination of M/M chimeric RNAs (Non-M/M); and after further retaining chimeric RNAs with frequency > 4 (Non-M/M-Recurrent). The distribution of each classifier changes after application of each filter (Figure 3). Most predicted chimeric RNAs in All-GTEx fall within the M/M (68%) and inter-chromosomal (80%) classifications, while the E/E (4%) and read-through (4%) chimeric RNAs exhibit considerably lower representation (Figure 3A). Consistently, we found that the majority of predicted chimeras (∼57.51%) were both inter-chromosomal and M/M (Supplementary Figure S1). As we move from All-GTEx to Non-M/Ms and further to Non-M/M-Recurrent datasets, the percentage of E/E chimeras increased from 4% to 14%, then to 21%. Similarly, read-through chimeras changed from 4% to 7%, then to 22%, indicating that E/E and read-through events are enriched as more stringent filters were applied (Figure 3A–C). The distribution of fusion protein coding categories does not change significantly when different filters were applied (Figure 3). The number of chimeric RNAs is also plotted based on their frequency (Figure 3D).

We then examined the canonical splicing sequences at the 5′ and 3′ junctions. Specifically, we searched for canonical AG/GT sequence at the 5′ junction (AG before the junction, and GT after the junction); as well as canonical AG/G sequence at the 3′ junction (AG before the junction, and G after the junction). Not surprisingly, for the 5′ junction, a higher percent E/E and E/M chimeric RNAs have the canonical splicing donor sequence AG/GT (42 and 15.5%, respectively); Whereas the M/E and M/M chimeric RNAs have a lower percent (6 and 1.7%, respectively). For the 3′ junction, E/E and M/E categories have more AG/G sequence (42.5 and 34.8%, respectively), and E/M and M/M have less (10 and 9.4%, respectively) (Figure 3E). When we examined the recurrent chimeric RNAs, the percentage of chimeric RNAs harboring the canonical splicing sequences increased, especially for the M/E category, but not much for the others. In any situation, chimeric RNAs in the M/M category has the least number of canonical splicing sequences.

**Figure 4.**
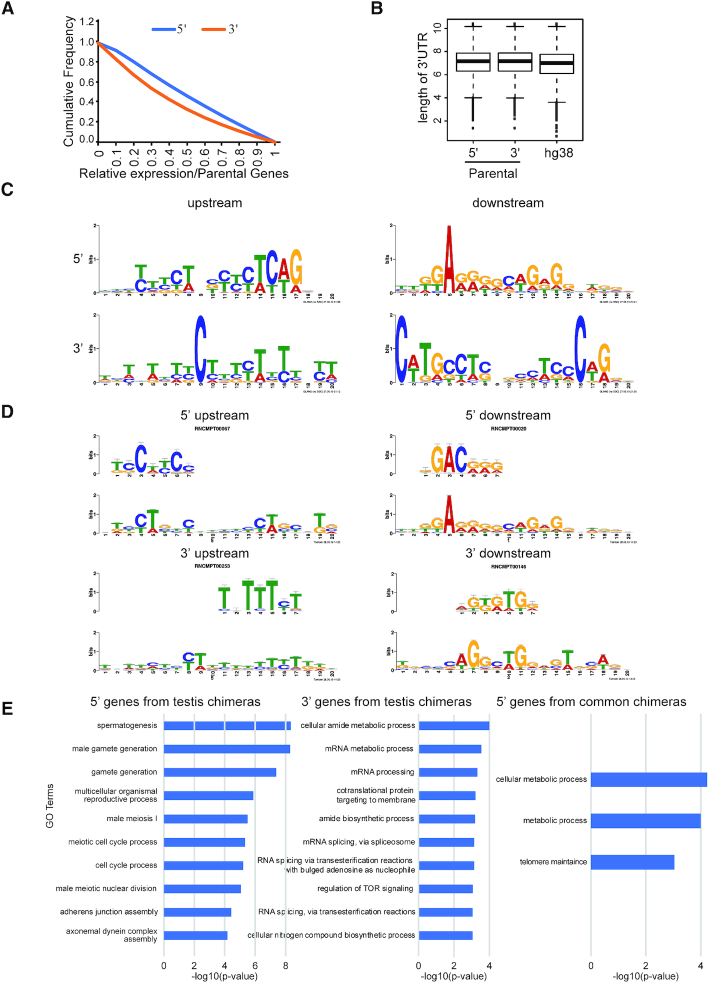
Characterization of chimeric RNAs and their parental genes. (**A**) Cumulative frequency distribution of relative chimeric transcript to parental gene expression. (**B**) Boxplots for 3′ UTR length of 5′ parental genes, 3′ parental genes and all annotated genes in the hg38 genome. (**C**) Sequence logo of the most enriched motifs identified in upstream and downstream sequences from the chimeric junction. (**D**) Example of one of the most enriched RNA binding motifs scanned by Tomtom. (**E**) Gene Ontology molecular process terms for parental genes of testis-specific chimeras and common chimeras present in all 53 tissues. No significant GO terms were found for the 3′ genes of chimeras common to all tissues.

Next, we aimed to assess the distribution of chimeric RNA-forming parental genes throughout the genome. We plotted the relation between the total number of annotated genes in hg38 and the number of chimera-forming genes on each chromosome. We observed a strong correlation, suggesting that the parental genes are distributed consistently throughout the genome (Supplementary Figure S2). We then examined the expression of chimeric RNAs relative to their parental genes, and found that almost half of the total chimeras (All-GTEx set) are expressed at ≥50% level relative to their 5′ parental genes. With respect to the 3′ parental genes, 45% of the chimeras are expressed at ≥50% level (Figure 4A). Relative expression of chimeras from later filtering stages (Non-MM and Non-MM-Recurrent) also followed a similar pattern (Supplementary Figure S3).

As the 3′ UTR is the primary site for microRNA (miRNA) regulation, and the structure of a typical chimeric RNA joins the 3′ UTR from the 3′ gene to the 5′ transcript, we are interested in investigating whether forming chimeric RNAs may result in differential regulation by microRNAs. Indeed, it has been reported that forming the PAX3-FOXO1 chimera allows human cells to escape the regulation of miR-495 on its parental PAX3 gene (49). To study genome-level changes of miRNA regulation, we evaluated the length of the 3′ UTR as a proxy. Interestingly, the average length of the parental gene 3′ UTR was longer than the average length of the 3′ UTR of all the genes in the hg38 genome (*P*-value 2.2e-37, Mann–Whitney U test) (Figure 4B and Supplementary Figure S4). However, we did not observe any statistical difference between the average length of the 3′ UTR of 5′ and 3′ parental genes of chimeric RNAs (*P*-value 0.75, Mann–Whitney U test) (Figure 4B and Supplementary Figure S4).

We then searched for DNA motifs surrounding the chimeric junction sites. We obtained 200 bp sequences upstream and downstream of fusion junction sites of both 5′ and 3′ parental genes. We used the MEME motif discovery tool (41) and GLAM2 (40) to look for sequence motifs enriched in these fragments. The motifs presented in Figure 4C are the highest scoring for upstream and downstream sequences of 5′ and 3′ genes.

Further, we used the Tomtom tool (42) aligning with the motif from GLAM2 (40) to assess the potential for these enriched motifs as sites for RNA binding proteins. Several motifs were identified in the upstream and downstream regions of the 5′ and 3′ parental genes of the chimeric RNAs (Figure 4D). For example, in the 5′ upstream region, motifs such as SRSF9, SRSF10, ENOX1, PTBP1, PCBP2 were identified. In the 5′ downstream region, motifs such as FXR2, PCBP1, Tb_0217, PTBP1 and SRSF10 were identified. In the 3′ upstream region, motifs such as Tb_0253, SART3, PABPC1, PABPC4 and IGF2BP3 were identified. In the 3′ downstream region, motifs such as CG7804, SRSF9, SRSF2, PCBP1 and PCBP3 were identified. (Figure 4D and Supplementary Figure S5). Among them, many motifs including SRSF9, PTBP1, SART3, Tb_0253 and PABPC1 were also found in our previous study (14).

### GTEx chimeric peptides

Chimeric RNAs in normal physiology have the potential to produce chimeric proteins (50,51). Due to the lack of proteomic data from GTEx, we downloaded raw mass spectrometry data for colonic tissue from the Clinical Proteomic Tumor Analysis Consortium (CPTAC) (30). We probed this dataset for chimeric peptides predicted within the Non-M/M-Recurrent grouping in colon tissues. We identified a total of 25 PSMs after applying a cutoff of *q* < 0.05, which map back to a total of 15 unique peptides (Supplementary Table S4). These 15 chimeric peptides map back to a list of chimeric RNAs. We also performed tblastn with identified chimeric peptide sequence queries against human translated RefSeq RNA database (35,36) as an additional step to rule out regular transcripts that may produce same peptides as chimeric RNAs. Four chimeric peptides did not have any hits in RefSeq, while eight peptides had match with only one side of the junction sequences. In summary, 12 peptides were identified that are likely products of chimeric RNAs (Supplementary Table S4).

One of these chimeric peptides, SLC39A1-CRTC2, was previously identified by our group in MCF10A (breast) cell lines (14). In this study, we found this chimeric RNA in multiple tissues including breast and colon (sigmoid and transverse), and confirmed by the above normal colon Mass spectrometry data. Interestingly, several are not predicted to produce in-frame chimeric proteins by FusionCatcher software (38), suggesting that these may encode truncated peptides or make use of alternative reading frames.

### Gene ontology prediction

We identified 40 chimeric RNAs common to all 53 tissues (Supplementary Table S5). Gene ontology enrichment analysis (GO) (39) revealed significant enrichment in processes such as ‘cellular metabolic process’ (69%), ‘metabolic process’ (72%) and ‘telomere maintenance’ (8%) for the 5′ genes. These processes are fundamental to all cells and explains why these chimeras are commonly present in all tissues. No significant enriched GO terms were found in the 3′ genes (Figure 4E). We also extended our analysis of GO analysis for molecular function and cellular component terms for the common chimeras and found terms ‘small ribosomal subunit rRNA binding’ and ‘intracellular organelle part’, respectively.

We then compiled a list of tissue-specific recurrent chimeras and observed that these are most common in testis (566) followed by whole blood (117) and skeletal muscle (52) (Supplementary Table S6). GO analysis for the 5′ parental genes of the tissue-specific chimeras in testis, whole blood and skeletal muscle revealed normal processes specific to each respective tissue. For example, processes such as ‘spermatogenesis’ (7%), ‘gamete generation’ (7%) and ‘male meiosis I’ (7%), were enriched in the 5′ parental gene of the testis-specific chimeras (Figure 4E and Supplementary Figure S6).

### Validation and functional assessment for a subset of chimeras

**Figure 5.**
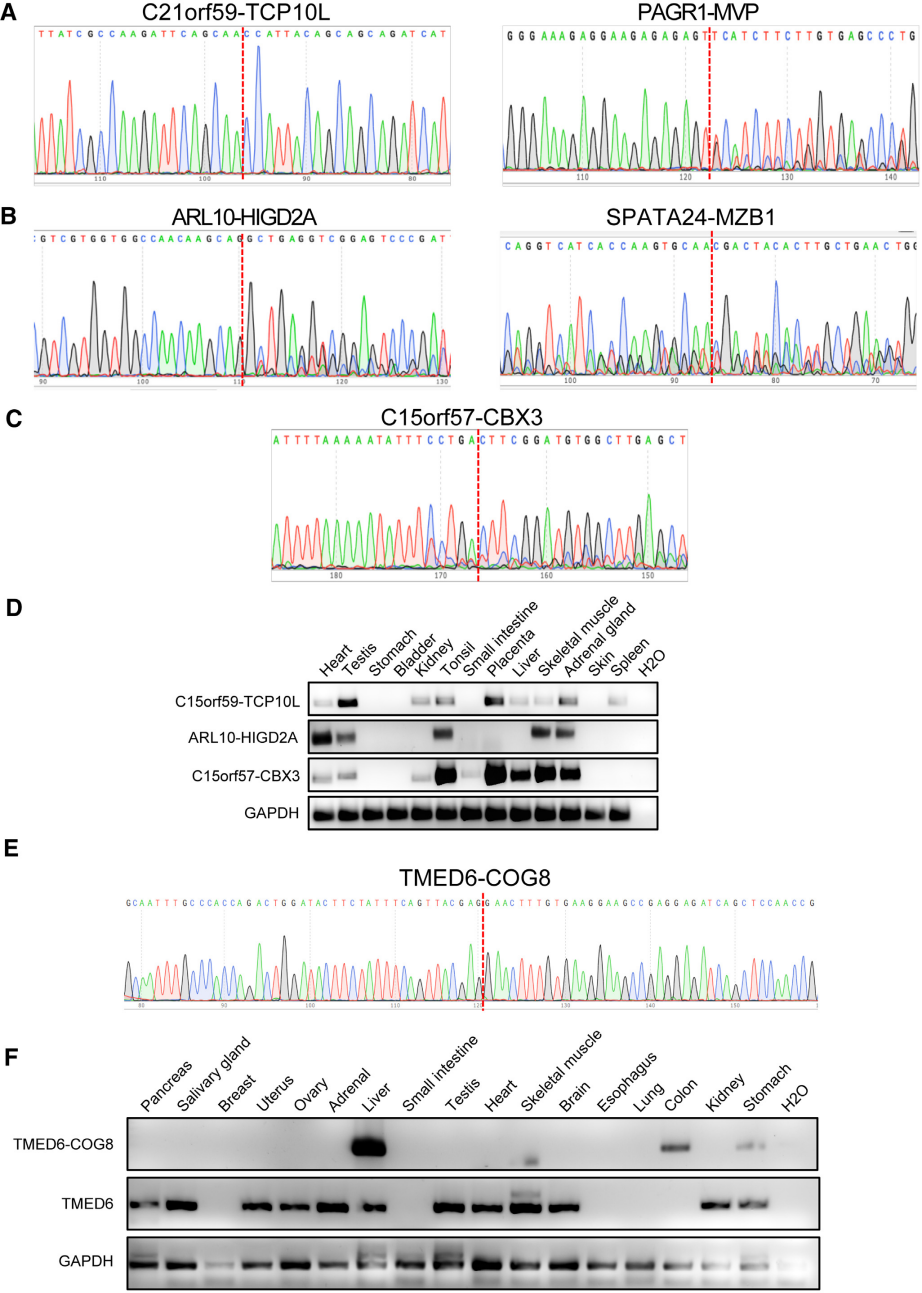
Identification and profiling of chimeric RNA candidates. Sanger sequencing of chimeric RNA candidates from read-through (**A**), intra-others (**B**) and inter-chromosomal (**C**). The chimeric RNA expression in human normal tissues was examined by qRT-PCR and followed by gel electrophoresis (**D**). Tissue specific fusion RNA, *TMED6-COG6*, was found only in liver, colon and stomach by qRT-PCR, whereas the wild-type parental gene *TMED6* was found in a different list of tissues (**E** and **F**). *GAPDH* was used as internal control.

We selected candidate chimeric RNAs from each parental gene combination class, designed primers flanking the fusion junction. Based on the chimeric RNA junction classes (E/E, E/M and M/E), fusion types (read-through, intra-other and inter-chr) and their frequencies in GTEx samples, we chose 38, 30 and 39 candidates from read-through, intra-other and inter-chr groups respectively for validation. Sanger sequencing was used after RT-PCR to confirm the chimeras with >20 bp of DNA sequence on both sides of the junction (Figure 5A–C). Twenty-one and eight were validated from read-through and intra-other, respectively. However, only one chimeric RNA, *C15orf57-CBX3* was confirmed from the inter-chr group (Supplementary Figure S7). The relatively lower validation rate than our previous study (47) is partly due to the fact that not the same samples used for discovery were available for validation. We then examined the expression of the chimeras across a panel of normal tissues. Consistent with GTEx prediction, *C21orf59-TCP10L, ARL10-HIGD2A* and *C15orf57-CBX3* were detected in multiple tissues (Figure 5D). In contrast, *TMED6-COG8* was only detected in a few tissues (Figure 5E and F). Interestingly, the chimera's expression does not follow the pattern of the wild-type parental gene, *TMED6* (Figure 5F), while wild-type *COG8* was undetected in these samples.

**Figure 6.**
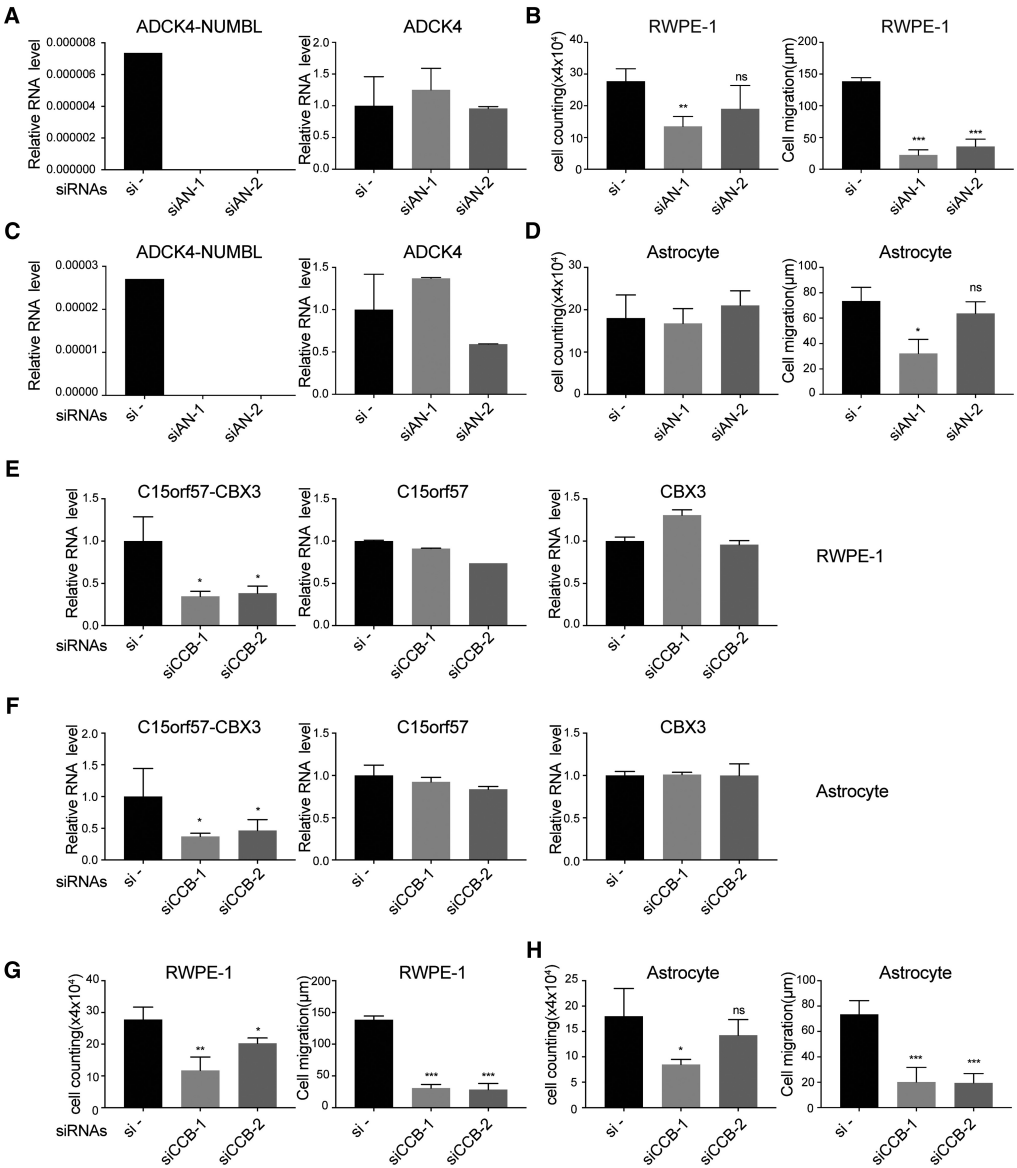
Knockdown of *ADCK4-NUMBL* and *C15orf57-CBX* decreased cell proliferation and/or cell motility in non-cancerous cells. (**A**) Two siRNAs specifically knocked down the fusion RNA *ADCK4-NUMBL* in RWPE-1, with no significant effect on the wild-type parental *ADCK4*. Wild-type *NUMBL* was too low to be detected. (**B**) In RWPE-1 cell line, cell proliferation was measured by cell counting (left), and cell motility was measured by wound healing assay (right). (**C**) Two siRNAs specifically knocked down the fusion RNA *ADCK4-NUMBL* in astrocytes, with no significant effect on the wild-type parental *ADCK4*. (**D**) In astrocytes, cell proliferation was measured by cell counting (left), and cell motility was measured by wound healing assay (right). (**E** and**F**) Two siRNAs specifically knocked down the chimeric RNA *C15orf57-CBX3* in RWPE-1 and astrocyte, with no significant effect on the wild-type parental *C15orf57* and *CBX3*. (**G** and **H**) Cell proliferation was measured by cell counting (left), and cell motility was measured by wound healing assay (right) in RWPE-1 and astrocyte respectively.

We then focused on studying the functional relevance of two chimeric RNAs, *ADCK4-NUMBL* and *C15orf57-CBX3*. *ADCK4-NUMBL* is a read-through chimera, a likely product from *cis*-splicing between adjacent genes (*cis*-SAGe). On the other hand, *C15orf57-CBX3* is an inter-chromosomal chimeric RNA, likely formed via trans-splicing. We selected these two chimeras, because both were detected in multiple tissues suggesting that they play some basic function that may be important across cell types. Two siRNAs were used to specifically target *ADCK4-NUMBL* in RWPE-1 cells (Figure 6A). Each siRNA dramatically knocked down the fusion RNA without significant effect on the wild-type parental transcript of *ADCK4*. The RNA level of wild-type *NUMBL* was too low to be detected in RWPE-1 cells. We observed reduced cell proliferation rate and significant cell motility reduction, when *ADCK4-NUMBL* was knocked down with the two siRNAs (Figure 6B). Similarly, the two siRNAs dramatically knocked down the expression of the chimera in astrocyte cells, with little effect on the wild-type *ADCK4* (Figure 6C). Different from the result from RWPE-1, we observed some reduction of cell migration when the chimera was silenced in astrocytes, while no significant change in cell proliferation was seen (Figure 6D). These results support a basic maintenance role of *ADCK4-NUMBL*, and suggest that some effects may be more cell type specific.

For the inter-chromosomal chimeric RNA, *C15orfCBX3*, the second exon of *C15orf57* (*CCDC32*) on chromosome 15 is fused with the first exon of *CBX3* on chromosome 7. We designed two siRNAs (siCCB-1 and siCCB-2) to specifically knock down the chimera in RWPE-1 and astrocytes. In both cell lines, siCCB-1 and siCCB-2 reduced the fusion RNA level with high specificity, with no significant changes to either wild-type parental gene (Figure 6E and F). Importantly, silencing *C15orf57-CBX3* resulted in significantly decreased cell proliferation and cell motility in RWPE-1 and astrocytes (Figure 6G and H).

### Overlap between GTEx chimeras and the database of cancer chimeras

**Figure 7.**
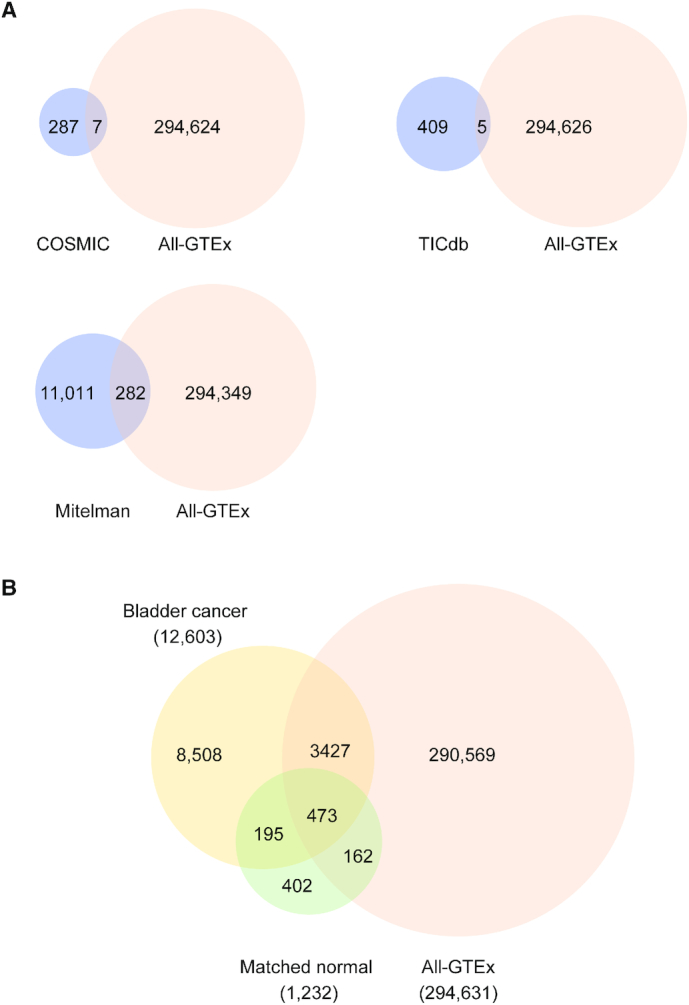
Overlaps between GTEx and cancer fusions. (**A**) Venn diagram showing common chimeras (gene pairs) among the All-GTEx set and three different databases (COSMIC, TICdb, Mitelman) of cancer fusions. (**B**) All-GTEx set and chimeras detected from TCGA bladder cancer and normal matched bladder tissues (matched normal).

Several chimeric RNAs thought to be specific to cancer were reported to be present in normal cells (14,18–19,53). Therefore, we suspected that some chimeras compiled in cancer databases may also be present in the GTEx non-diseased samples. Indeed, we found that several cancer chimeras listed within COSMIC (27), TICdb (29) and the Mitelman Database of Chromosome Aberrations and Gene Fusions in Cancer in the Cancer Genome Anatomy Project (54) were also present in our predictions (Figure 7A, and Supplementary Tables S7–10). Apart from gene-pairs, we also examined chimeric breakpoints in COSMIC database (27), which stores the breakpoint positions of the chimera with respect to mRNA sequence of the parental gene. We first converted the breakpoint position to genomic coordinates and compared with chimeric RNA junction coordinates in GTEx. Out of seven common chimeras between COSMIC and GTEx, we identified three chimeras (*BCR-ABL1*, *SLC45A3-ELK4* and *DHH-RHEBL1*) with the exactly same coordinates (i.e. same isoform).

Interestingly, the classic *BCR-ABL1* chimera found in most patients with chronic myelogenous leukemia (CML) (52,55–56), was detected in one skeletal muscle sample (Supplementary Tables S7–9). The detected chimera joins exon 14 of *BCR* to exon 2 of *ABL* (e14e2). This is surprising, as this form of *BCR-ABL* is a well-characterized cancer fusion in leukemia. It is possible that some contamination occurs during library preparation, and RNAs from one of the leukemia cell line, K562 or KU812 used as positive controls for GTEx were mixed in the skeletal muscle sample. However, the identification of *BCR-ABL* in the muscle sample is supported by four spanning reads, and three split reads, arguing against the possibility of a low-level contamination. Additionally, *SLC45A3-ELK4*, a transcriptional read-through chimeric RNA, which is commonly reported in prostate cancer, was also found in GTEx prostate, brain, and artery samples (Supplementary Tables S7–9). In prostate cancer, two isoforms of *SLC45A3*-*ELK4* (e1e2 and e4e2) have been reported (25,57–61), and the expression of the former has been shown to correlate with Gleason score (57,60). Interestingly, the predicted isoforms found in our study vary by tissues. We found e5e2 *SLC45A3-ELK4* in prostate, e4e2 and e1e3 in brain and e1e2 in artery (Supplementary Table S7–9), indicating that some isoforms of physiological chimeric RNAs may be aberrantly expressed in cancer.

To demonstrate the value of GTEx dataset as a resource for chimeric RNAs in normal physiology, we used it to filter predictions from the TCGA bladder cancer study (62). In total, the TCGA bladder cancer study contains 414 tumor samples and 19 matched normal samples. EricScript software was used to predict 19 547 unique chimeric RNAs from all 433 samples (63). A total of 12 603 gene pairs were found in cancer samples, and 1232 gene pairs in the matched normal samples (Supplementary Tables S10 and 11). Using chimeric RNAs from matched normal samples as a control dataset to filter out chimeras identified in cancer samples, we were able to eliminate 668 gene pairs, which represent ∼5% of total gene pairs from the list of cancer chimeras. On the other hand, using chimeras from GTEx as a control dataset, we eliminated 3900 (∼31%) gene pairs from the list of cancer chimeras (Figure 7B). Out of 668 chimeras filtered out by the matched control dataset, 473 were already represented in the GTEx dataset. Thus, the matched normal control set filtered only a small fraction of chimeras that were not encompassed by the GTEx predictions (1.5%). On the other hand, the 1.5% (195) of chimeras that are in both bladder cancer and matched normal samples, but not in GTEx, may represent some early molecular events during tumorigenesis, and warrant further investigation rather than elimination.

## DISCUSSION

Chimeric RNAs produced by chromosomal rearrangement are common features of neoplasia. On the other hand, chimeric RNAs detected in normal tissues and cells such as the ones we detected in GTEx are presumably produced in the absence of chromosomal rearrangement. Indeed, we examined 20 candidate chimeric RNAs, downloaded whole-genome sequencing data for the corresponding samples and detected no evidence of chromosomal rearrangement (examples shown in Supplementary Figure S8). Such chimeric RNAs provide an additional means for expansion of the functional genome without a concordant increase in the number of genes. Chimeras commonly present in many different tissues may represent a set of functional entities involved in fundamental cellular mechanisms common to all cells. We have shown that cell mobility and proliferative viability suffer in the absence of *C15orf57-CBX3* or *ADCK-NUMBL* and provided a listing of 38 additional predicted candidates which may possess similarly important functionality. We have shown that chimeric RNAs also have the ability to form chimeric proteins, and candidate chimeric peptides across junction can be identified using proteomics data 12. More studies on the functionality of chimeric proteins, including the efforts to map the chimeric protein–protein interactions (64,65), are warranted.

When we examined the canonical splicing junction sequence, we found that chimeric RNAs belonging to the M/M category have the lowest percent harboring such sequences. This and their lower experimental validation rates based on our previous study (47) support the notion that at least a large percent of them may represent artifacts during library construction (14). Therefore, we decided to filter them out for downstream studies.

We randomly selected over 100 chimeric candidates from different categories of junction classes (E/E, E/M and M/E), fusion types (read-through, intra-other and inter-chr) and their frequencies in GTEx samples for experimental validation. We had higher validation for read-through, and frequent chimeras. Overall the validation rate is low compared to another study we conducted previously (47). This could be due to the following reasons. (i) In our previous study, exact same RNA samples were used for RNA-seq and downstream validation, whereas here the same GTEx samples that have the RNA-seq data are not available for experimental validation. This is a more serious issue for less frequent chimeric RNAs than the frequent ones. (ii) Related to the first reason, we only used a small number of normal tissues in validation, and the heterogeneity of tissues complicates the validation. (iii) Different software tools were used between the two studies.

Several other databases have accumulated number of chimeric RNAs, including ChiTaRS (66), ChimerDB (67,68), TICdb (29), although most concentrated on cancer samples. We downloaded the ChiTaRS 3.1 dataset, and found 167 common chimeric RNAs between GTEx and ChiTaRS (Supplementary Table S12).

We also found 908 common gene pairs between the current study and our previous one (14). Compared with the previous study of chimeric RNAs in non-diseased tissues (14), we now present a far more comprehensive representation of normal chimeric RNA expression. Further, GTEx provides a robust panel of tissues, which fills gaps from our previous study in tissues such as adipose, ovary, prostate, which were under-represented due to a limited number of samples. Out of 30 chimeric RNAs experimentally validated previously, a total of 21, including *C15orf57-CBX3*, were also identified in this study. In both studies, we observed similar enrichment of read-through and E/E chimeric RNAs as we subjected the datasets to more stringent filters. Further, in capturing a more complete landscape of chimeric RNA expression in non-diseased tissues, we detected a number of transcripts also listed within the COSMIC (27), Mitelman (28), and TICdb (29) databases. These findings indicate that greater emphasis must be placed on validation of chimeric transcripts as biomarkers, as detection in cancer tissues/cells is insufficient to the claim that they are unique to cancer.

It is a common practice to use cancer-matched normal tissues as controls. However, these controls have significant limitations in sample size and RNA-seq data availability. Additionally, normal margins may be under the influence of a ‘field effect’ (41) and may harbor early tumorigenic events. Thus, using normal margins may not properly eliminate false positive nor false negative discoveries. In contrast, GTEx provides a large collection of samples, which more closely reflect the normal physiology of these tissues. We envision that this study will serve as a platform for further studies of chimeric RNAs in normal physiology and will be instrumental as a true normal baseline for assessment of chimeric transcripts in cancer.

## Supplementary Material

gkz1223_Supplemental_Files
