# Supplementary material for: The landscape of chimeric RNAs in non-diseased tissues and cells
Source: Nucleic Acids Res. 2020 Jan 22;48(4):1764–78. doi: 10.1093/nar/gkz1223 (PMC7038929; doi:10.1093/nar/gkz1223)
Supplement: gkz1223_Supplemental_Files [file gkz1223_supplemental_files.zip › SUPPLEMENTARY text.docx]

**SUPPLEMENTARY FIGURE LEGENDS**

**Figure S1. Distribution of chimeras belonging to different E, or M categories in (A) All-GTEx set and (B) read-throughs.**

**Figure S2. Correlation between the total number of genes on each chromosome and the number of genes forming chimeras for (A) All-GTEx set, (B) Non-M/M set, and (C) Non-M/M-Recurrent set.**

**Figure S3. Cumulative frequency of the relative expression of the chimeric transcripts to their parental genes for (A) Non-M/M set, and (B) Non-M/M-Recurrent set.**

**Figure S4. Comparison of the length of 3’ UTR of parental genes for (A) Non-M/M set, and (B) Non-M/M-Recurrent set with hg38 genome.**

**Figure S5. Additional RNA binding motifs identified by Tomtom tool using the best GLAM2 motif in the (A) 5’ upstream and 5’ downstream, (B) 3’ upstream and 3’ downstream regions of the Non-M/M-Recurrent chimeras.**

**Figure S6. GO terms for parental genes of additional tissue specific chimeras in (A-B) blood tissue and (C-D) muscle tissue.**

**Figure S7. The number of primers designed for chimeric RNAs validation, and the number of validated chimeras in each group.**

**Figure S8. IGV view of the genomic region covering the parental genes of three chimeric RNAs.** No split reads or evidence of deletion (in the cases of read-through chimeras) was observed.

**SUPPLEMENTARY TABLES**

**Table S1. List of primers used in this study.**

**Table S2. Number of samples, chimeras and their types in each tissue in GTEx. (#GTEx, #non M/M and #non M/M recurrent means number of chimeras in All-GTEx set, non-M/M set and non-M/M-recurrent set respectively.**

**Table S3. List of 15,979 chimeras (8262 unique gene-pairs) from GTEx noM/M recurrent set.**

**Table S4. Chimeric peptides identified from normal colon tissue and their corresponding chimera.**

**Table S5. List of 94 common chimeras present in all 53 tissues in no M/M set.**

**Table S6. List of tissue specific recurrent chimeras.**

**Table S7. List of common chimeras among GTEx and COSMIC cancer chimeric database.**

**Table S8. List of common chimeras among GTEx and TICdb cancer chimeric database.**

**Table S9. List of common chimeras among GTEx and Mitelman cancer chimeric database.**

**Table S10.** **18,727 Chimeric RNAs (12,603 gene pairs) from TCGA bladder cancer samples.**

**Table S11.** **1,379 Chimeric RNAs (1,232 gene pairs) from TCGA bladder normal matched samples.**

**Table S12. List of common chimeras between GTEx and ChiTaRS-3.1 database.**
