# Supplementary figures and images for: The landscape of chimeric RNAs in non-diseased tissues and cells
Source: Nucleic Acids Res. 2020 Jan 22;48(4):1764–78. doi: 10.1093/nar/gkz1223 (PMC7038929; doi:10.1093/nar/gkz1223)

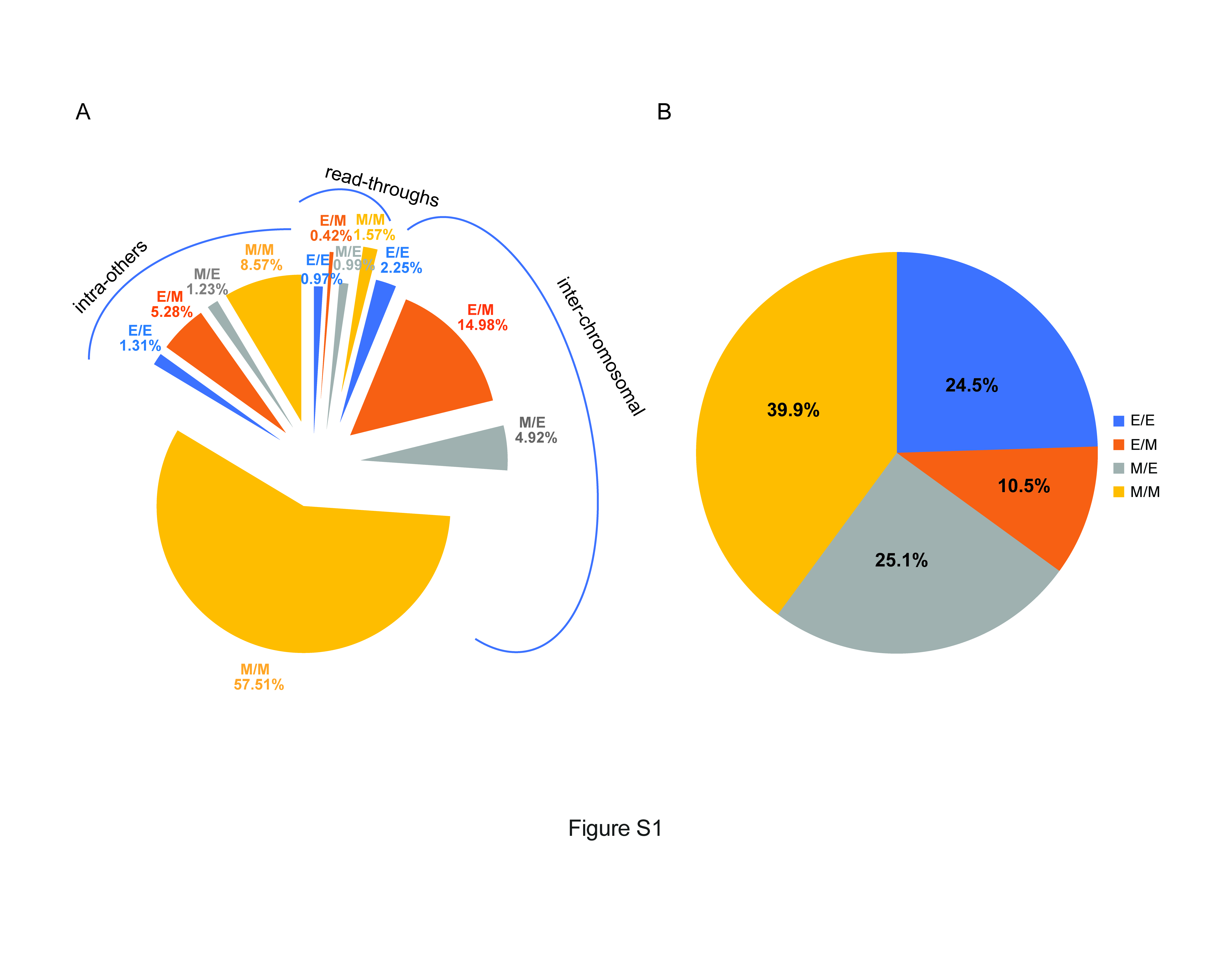

Supplement: gkz1223_Supplemental_Files [file gkz1223_supplemental_files.zip › Figure_S1.tif]

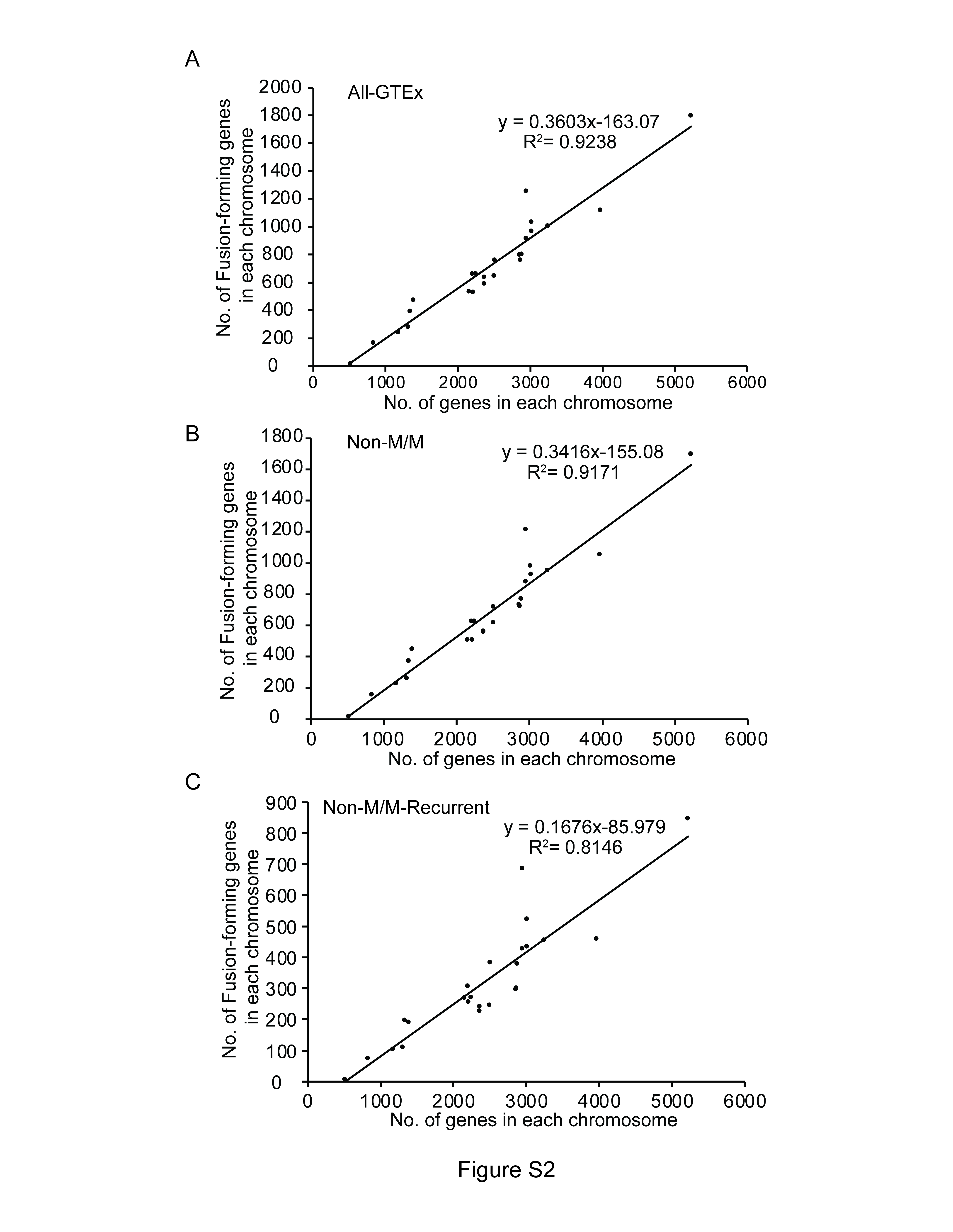

Supplement: gkz1223_Supplemental_Files [file gkz1223_supplemental_files.zip › Figure_S2.tif]

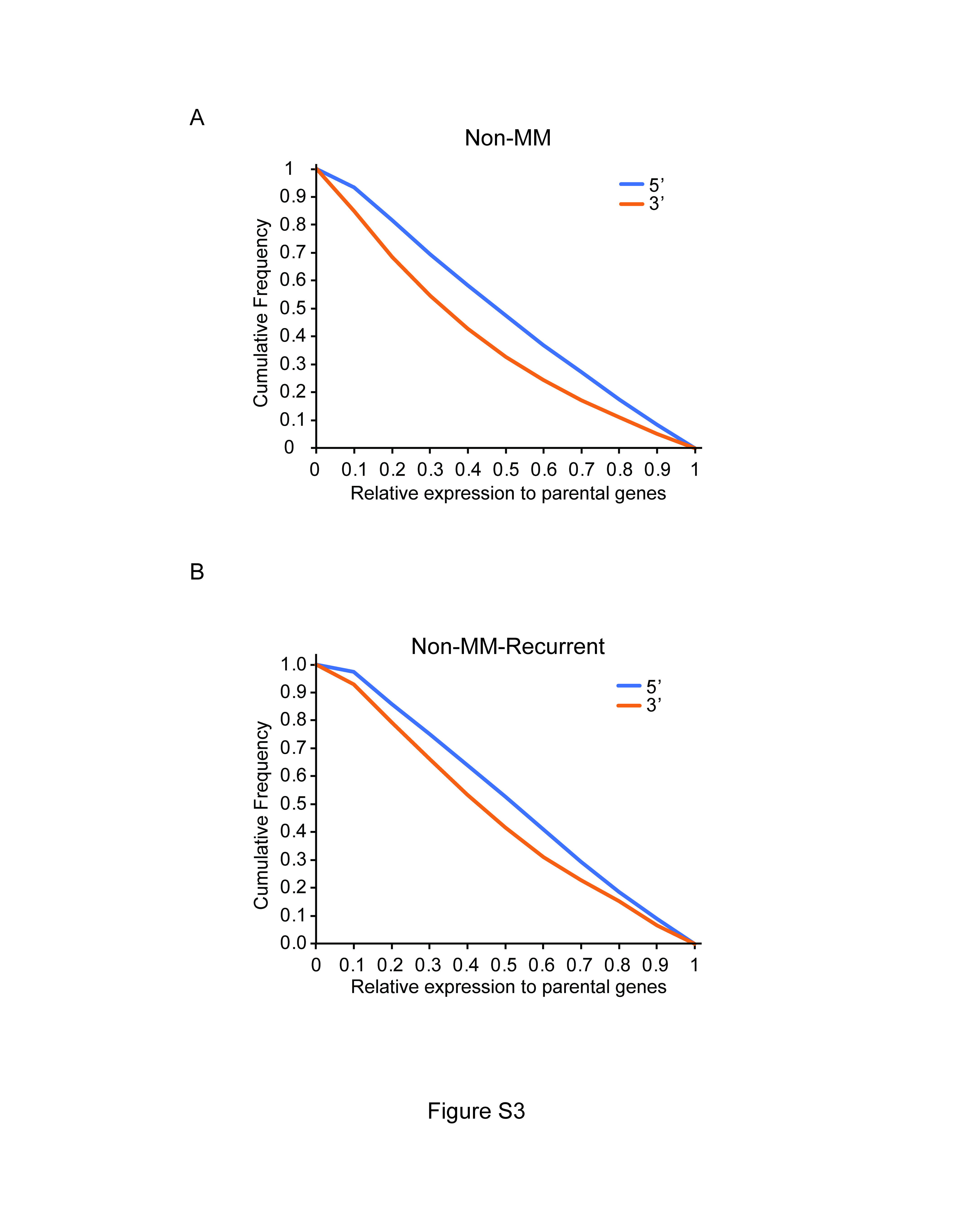

Supplement: gkz1223_Supplemental_Files [file gkz1223_supplemental_files.zip › Figure_S3.tif]

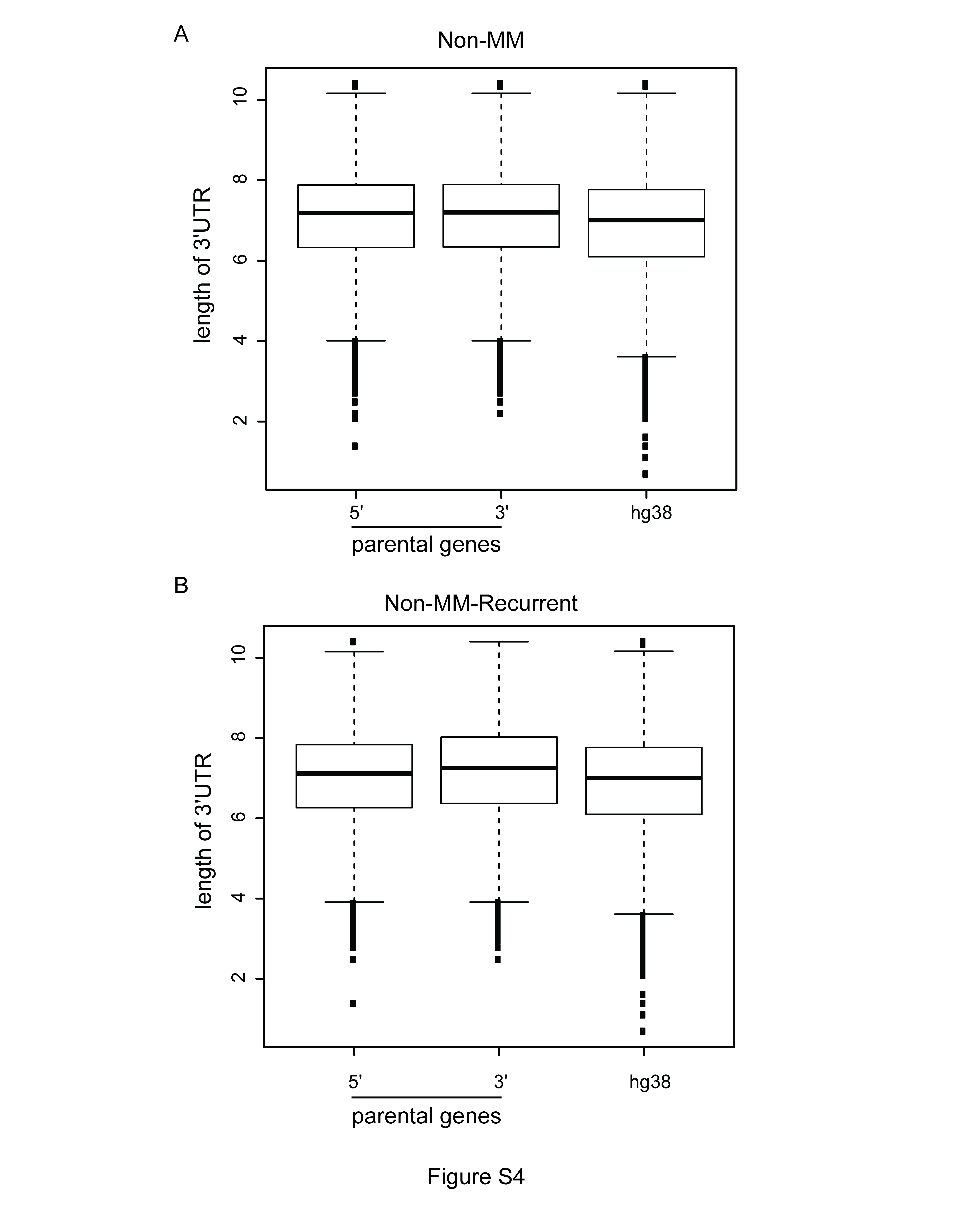

Supplement: gkz1223_Supplemental_Files [file gkz1223_supplemental_files.zip › Figure_S4.tif]

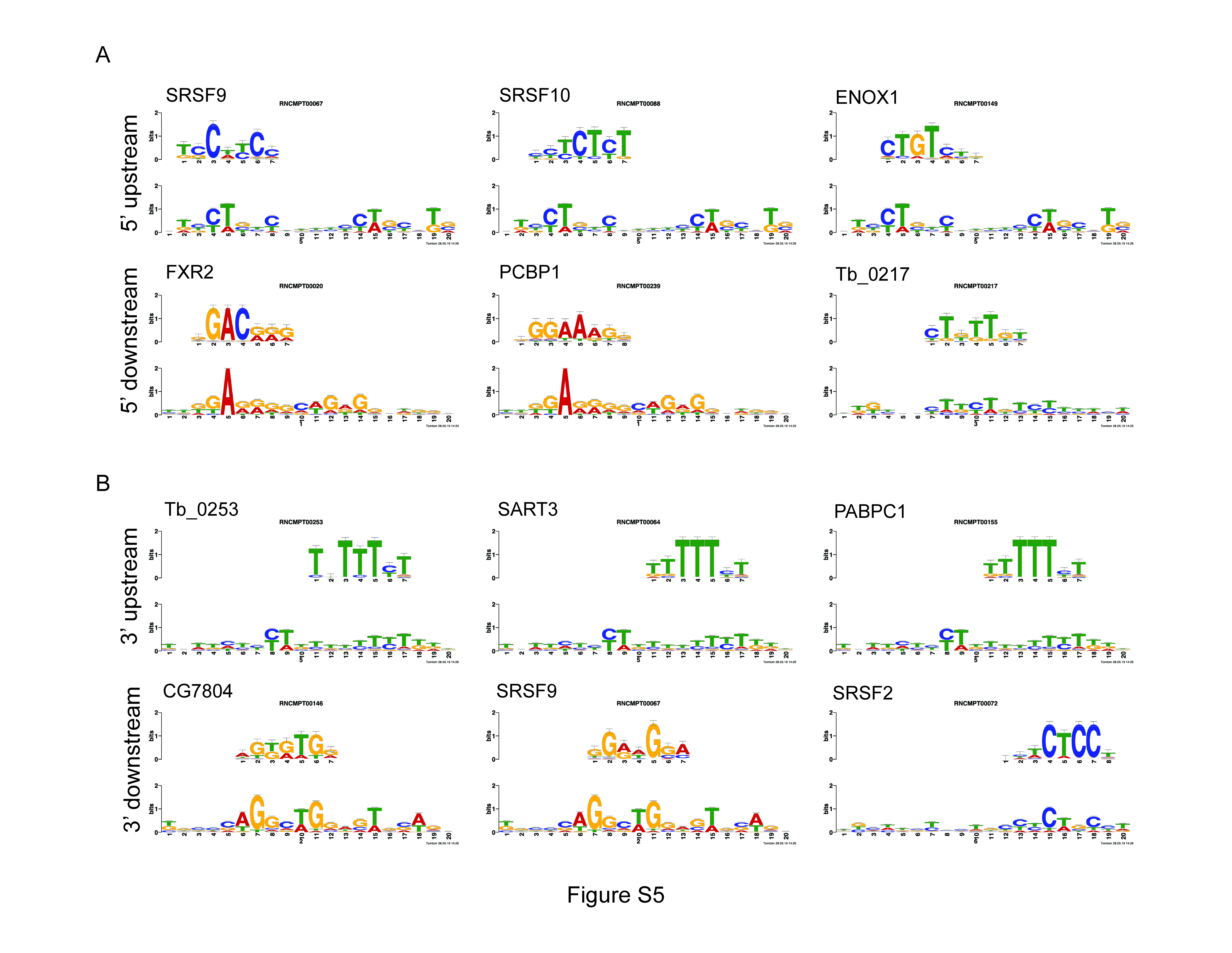

Supplement: gkz1223_Supplemental_Files [file gkz1223_supplemental_files.zip › Figure_S5.tif]

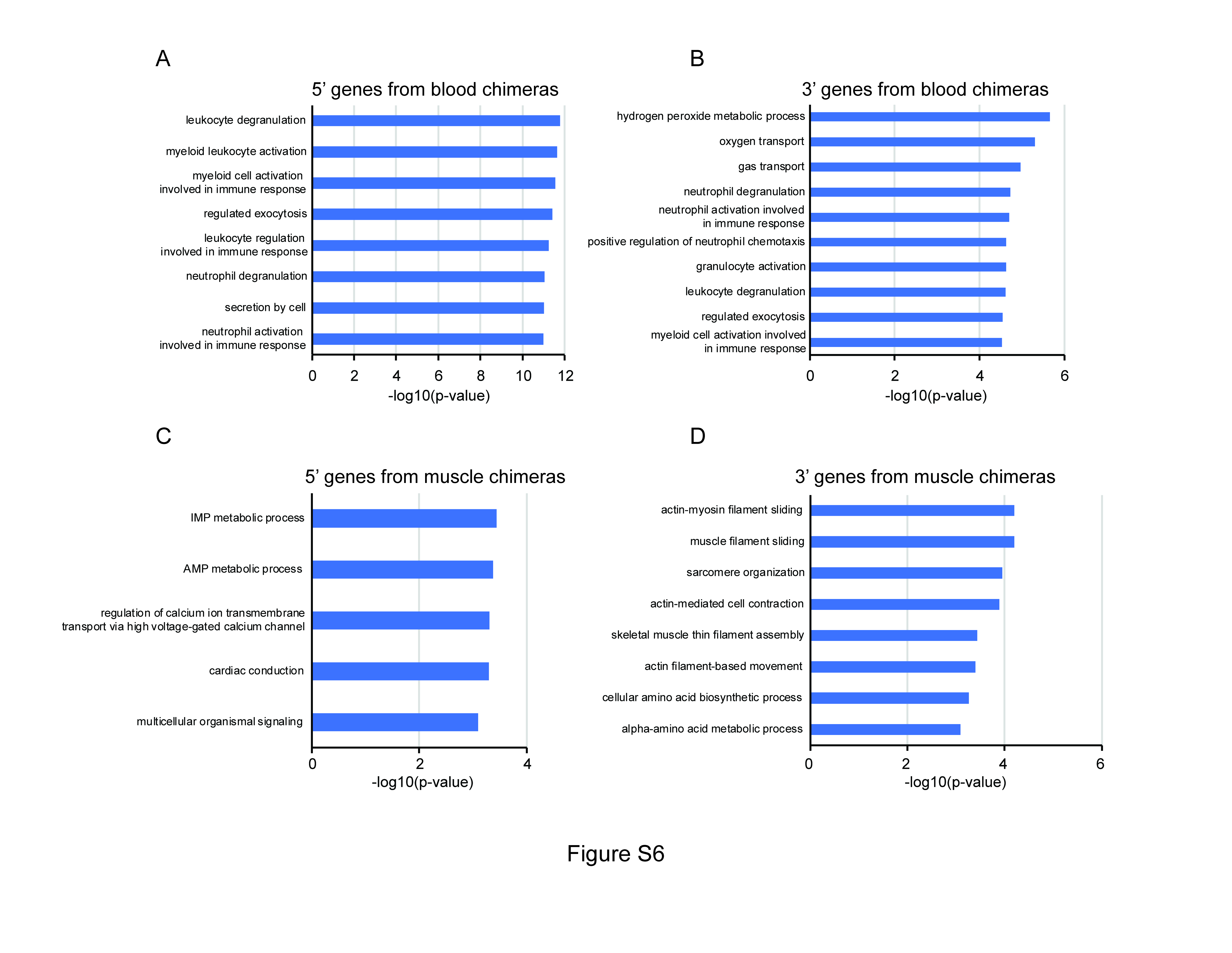

Supplement: gkz1223_Supplemental_Files [file gkz1223_supplemental_files.zip › Figure_S6.tif]

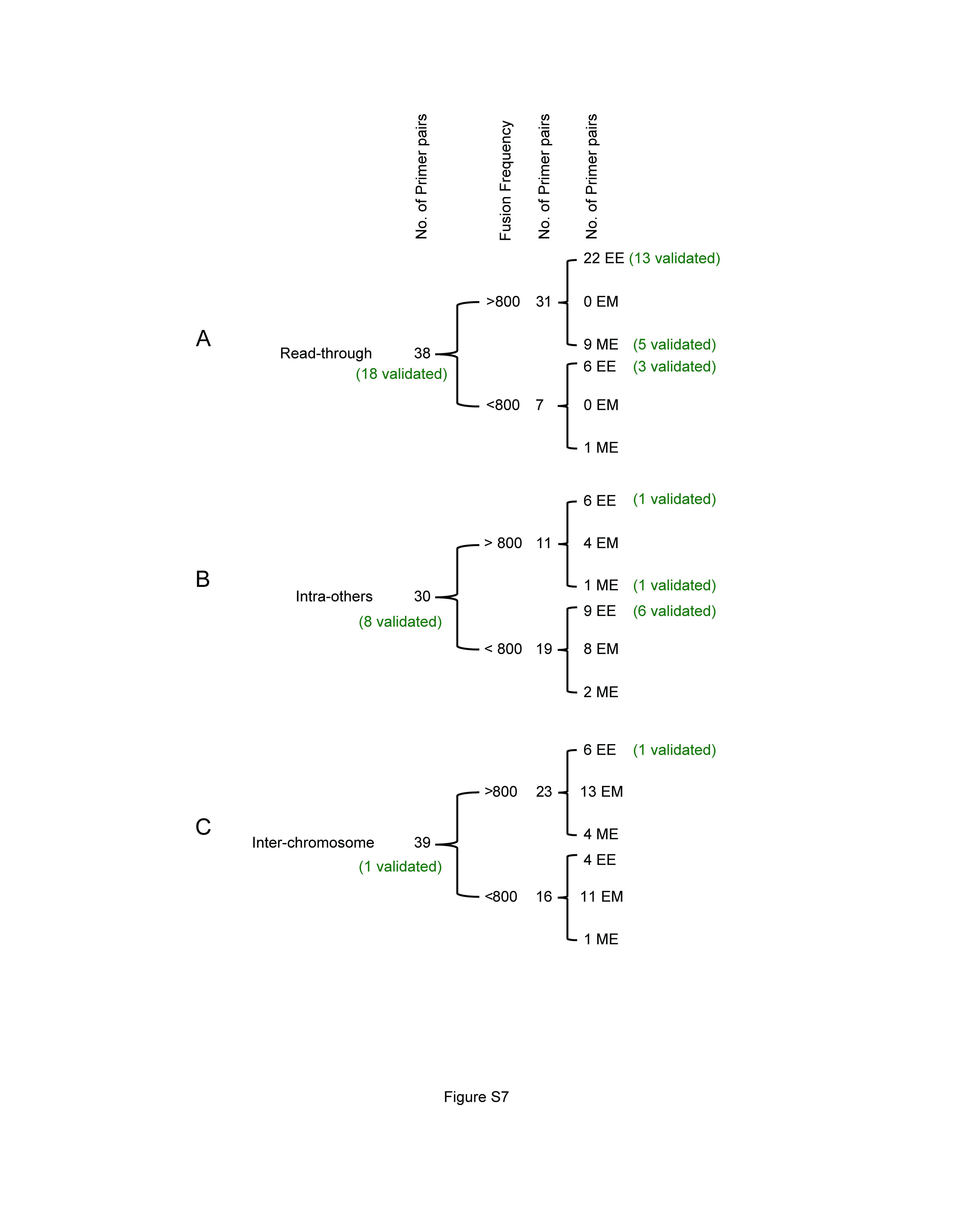

Supplement: gkz1223_Supplemental_Files [file gkz1223_supplemental_files.zip › Figure_S7.tif]

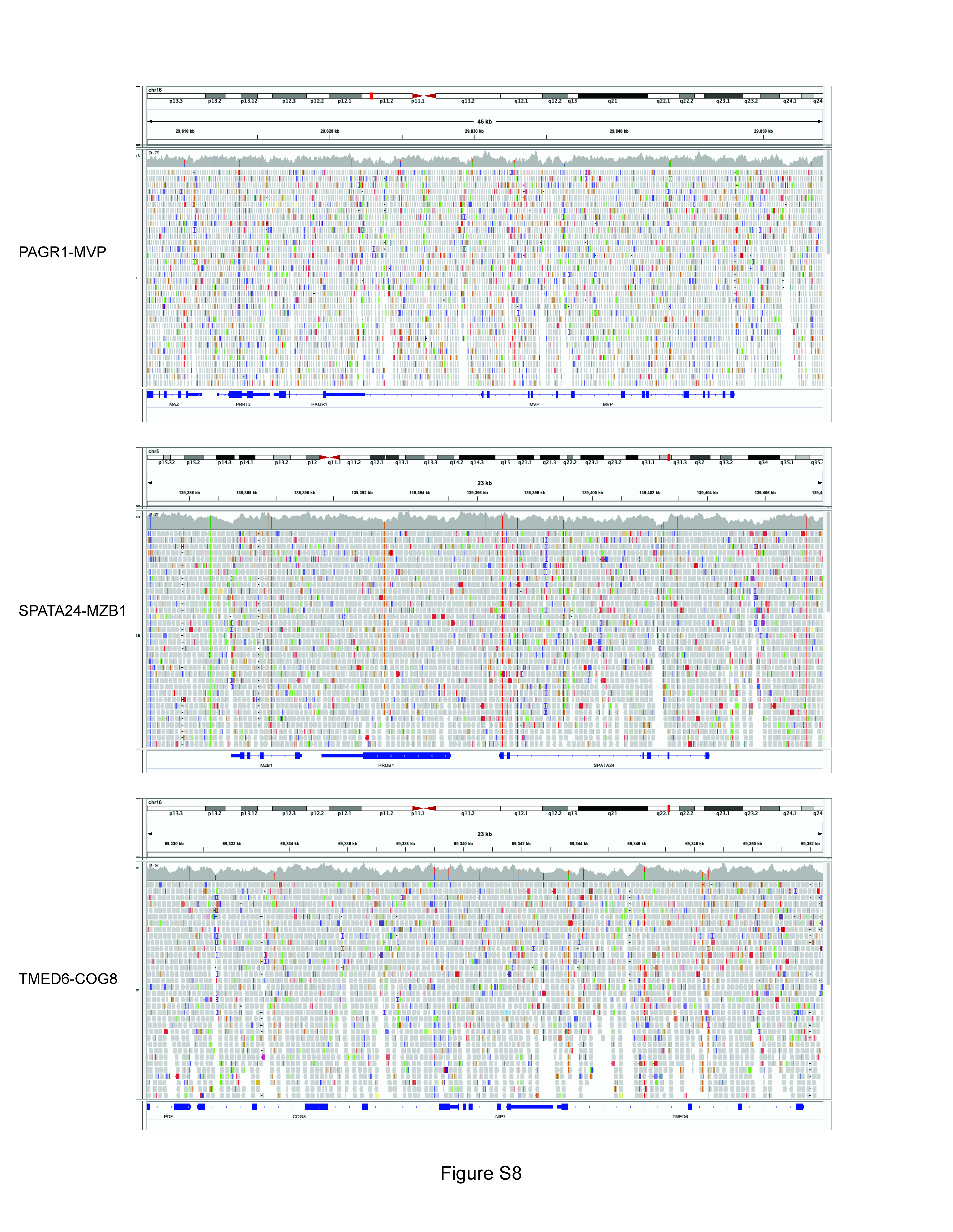

Supplement: gkz1223_Supplemental_Files [file gkz1223_supplemental_files.zip › Figure_S8.tif]
